# Critical evaluation of stents in coronary angioplasty: a systematic review

**DOI:** 10.1186/s12938-021-00883-7

**Published:** 2021-05-08

**Authors:** Joseph Robert Stevens, Ava Zamani, James Ian Atkins Osborne, Reza Zamani, Mohammad Akrami

**Affiliations:** 1 Medical School, College of Medicine and Health, Exeter, UK; 2 Medical School, University College London (UCL), London, UK; 3 Department of Mechanical Engineering, College of Engineering, Mathematics, and Physical Sciences, University of Exeter, Exeter, UK

**Keywords:** Coronary, Stent, Angioplasty, Percutaneous, Intervention, Revascularisation, Review

## Abstract

**Background:**

Coronary stents are routinely placed in the treatment and prophylaxis of coronary artery disease (CAD). Current coronary stent designs are prone to developing blockages: in-stent thrombosis (IST) and in-stent re-stenosis (ISR). This is a systematic review of the design of current coronary stent models, their structural properties and their modes of application, with a focus on their associated risks of IST and ISR. The primary aim of this review is to identify the best stent design features for reducing the risk of IST and ISR. To review the three major types of stents used in clinical settings today, determining best and relevant clinical practice by exploring which types and features of offer improved patient outcomes regarding coronary angioplasty. This information can potentially be used to increase the success rate of coronary angioplasty and stent technology in the future taking into account costs and benefits.

**Methods:**

Scientific databases were searched to find studies concerning stents. After the exclusion criteria were applied, 19 of the 3192 searched literature were included in this review. Studies investigating three major types of stent design were found: bare-metal stents (BMS), drug-eluting stents (DES) and bioresorbable stents (BRS). The number of participants varied between 14 and 1264. On average 77.4% were male, with a mean age of 64 years.

**Results:**

From the findings of these studies, it is clear that DES are superior in reducing the risk of ISR when compared to BMS. Conflicting results do not clarify whether BRS are superior to DES at reducing IST occurrence, although studies into newer BRS technologies show reducing events of IST to 0, creating a promising future for BRS showing them to be non-inferior. Thinner stents were shown to reduce IST rates, due to better re-endothelialisation. Scaffold material has also been shown to play a role with cobalt alloy stents reducing the risk of IST. This study found that thinner stents that release drugs were better at preventing re-blockages. Some dissolvable stents might be better at stopping blood clots blocking the arteries when compared to metal stents. The method and procedure of implanting the stent during coronary angioplasty influences success rate of these stents, meaning stent design is not the only significant factor to consider.

**Conclusions:**

Positive developments in coronary angioplasty could be made by designing new stents that encompass all the most desirable properties of existing stent technology. Further work is needed to investigate the benefits of BRS in reducing the risk of IST compared to DES, as well as to investigate the effects of different scaffold materials on IST and ISR outcomes.

## Background

In coronary artery disease (CAD), the coronary arteries are narrowed or blocked, impeding the supply of oxygen to the heart, resulting in damage to the heart muscle and its function. Approximately 125,000 people in the United Kingdom die each year due to CAD, and it is the leading cause of death globally [1]. Percutaneous coronary interventions (PCI) and coronary artery bypass grafts (CABG) are the main treatments for narrowed or blocked arteries due to CAD. CABG is a surgical procedure that entails a vein, usually grafted from the leg, at points above and below the level of occlusion, hence creating a ‘bypass’. However, this procedure is not as simple as it may sound, as differences in pressure values of stenosed arteries need to be considered [2]. PCI is an invasive procedure performed to widen blocked or narrowed coronary arteries, allowing increased blood flow, to resupply oxygen to the cardiac tissue [3]. Traditionally, PCI has been performed using balloon angioplasty alone. Balloon angioplasty is a technique in which a small balloon is delivered to the site of coronary artery occlusion. The balloon is then inflated, re-establishing blood flow. Early studies suggested that balloon angioplasty resulted in higher mortality rates compared to CABG; tissue lining the arterial wall (the endothelium), could become damaged, weakening the arterial wall and leading to re-narrowing of the artery (restenosis) [4]. This increased the risk of major adverse events, such as myocardial infarction and stroke [5].

### Bare-metal stents (BMS)

In 1986, Sigwart and Puel made major advances in PCI with the development of coronary bare-metal stents (BMS). BMS are cylindrical metal wire meshes that are expanded using balloon angioplasty and then remain in situ post-procedure. They are designed to keep the artery open after the initial balloon inflation [6]. BMS were developed to reduce the high frequency of restenosis after balloon angioplasty.

However, deploying a stent with a metal-based scaffold into an artery has since been found to be problematic in several ways. The interaction between bare-metal stent struts and endothelium can lead to endothelial damage, activation of the clotting cascade, platelet aggregation, and, potentially, formation of a blood clot (in-stent thrombosis or IST) [7]. Vascular smooth muscle cells (which provide structural integrity to the arterial wall) may also become damaged upon stent expansion, leading to vascular smooth muscle cell proliferation and migration to the surface of the endothelium as part of a process known as ‘neointimal hyperplasia’. Neointimal hyperplasia may reduce arterial lumen size and is thus increases the risk of in-stent restenosis (ISR) [8]. Patients who receive a BMS will be prescribed dual anti-platelet therapy (DAPT) for up to 12 months post-procedure. The drugs are usually aspirin and a drug that inhibits the key receptor for platelet aggregation (P2Y_12_); clopidogrel and prasugrel being the most common. This therapy is provided to help reduce the risk of in-stent thrombosis (IST) [9].

### Drug-eluting stents (DES)

Drug-eluting stents (DES) were first introduced in 1999 by Eduardo Sousa, to combat the high ISR risk of BMS. DES are metal-based stents with a durable-polymer layer, which elutes a drug after deployment [10]. The first drug used in DES was sirolimus, also known as rapamycin. Sirolimus is an immunosuppressive compound that inhibits the actions of mTOR, a kinase that promotes cell growth and proliferation. This inhibition is thought to reduce the proliferation of vascular smooth muscle cells, making it a suitable drug target to reduce ISR with metal stents [11]. Everolimus, zotarolimus, and umirolimus (biolimus) are all analogues of sirolimus which have been shown to reduce neointimal hyperplasia [12–14]. Paclitaxel, a well-known anti-cancer drug, has also been used in DES. It has been shown to inhibit vascular smooth muscle cell proliferation [15] and disturbs microtubule organisation, increasing the production of unstable microtubules, which reduces neointima formation, thereby reducing the incidence of ISR [16, 17]. Conflictingly, these drugs also inhibit the endothelial coverage of the stent struts (endothelialisation). This can lead to inflammation and platelet aggregation against the exposed stent struts, leading to IST formation (very late stent thrombosis) [18].

### Bioresorbable stents (BRS)

Bioresorbable stents (BRS) (also referred to as ‘biodegradable’ stents) were developed to combat the very late IST caused primarily by the presence of the metal stent struts. BRS are formed from biodegradable-polymers that form a strong radial scaffold. The ability of the stent to ‘disappear’ within 12 months of deployment reduces the risk of exposed stent struts arising, as with DES. BRS were designed so that the lesion in the arterial wall (atherosclerotic plaque) will have healed by the time the stent struts have degraded.

Poly-l-lactide is a biodegradable polymer commonly used in BRS as it degrades into lactic acid and so is metabolised by the body [19]. The everolimus-eluting bioresorbable stent (ABSORB) was the first of the commercially available BRS. ABSORBs performed well compared with DES; however, they were associated with a higher incidence of IST and myocardial infarction [20]. These increased risks appear to negate the potential long-term benefits of the BRS [20].

More recent developments in the area have produced the magnesium based BRS, DREAMS (drug-eluting absorbable metal scaffold) 2G (2nd generation) sirolimus-eluting Magmaris™ by Biotronik. This BRS lowers the incidence of IST dramatically, countering the main drawback of other BRS on the market, creating new potential for BRS treatments in the future. This was proven via a 12-month follow-up study on patients with the BRS implanted and a 3-year follow-up study on the same patients, showing that this BRS remains unproblematic in the long term. The patients were all deemed low risk, so the BRS is considered safe for this group only. Higher risk patients may not produce the same results. Refer to Table 1 for patient information and Table 2 for IST rates [21, 22]. The polymer-based structure is weaker than its metal counterpart, and there is therefore an increased risk of recoiling by the arterial wall, leading to ISR. Typically, the stent struts of BRS are wider and thicker than traditional metal stents, which affects the rate of endothelialisation of the stent as well as deliverability (see Table 3). A larger stent also requires a larger catheter profile, which subsequently requires a more accomplished clinician to complete the intervention successfully [23].

**Table 1:** Literature search terms (in quotation marks) used in the Ovid program

	Search terms	Number of papers
	Stent type	
1	“bare metal stent ti,ab” OR “BMS ti,ab”	23,059
2	“drug eluting stent ti,ab” OR “DES ti,ab”	1,232,392
3	“everolimus eluting stent ti,ab” OR “EES ti,ab”	13,972
4	“sirolimus eluting stent ti,ab” OR “SES ti,ab”	74,773
5	“zotarolimus ti,ab” OR “ZES ti,ab”	4866
6	“biolimus eluting stent ti,ab” OR “BES ti,ab”	11,993
7	“paclitaxel eluting stent ti,ab” OR “PES ti,ab”	34,967
8	“bioresorbable stent ti,ab” OR “bioresorbable scaffold ti,ab” OR “BRS ti,ab”	15,216
9	1 OR 2 OR 3 OR 4 OR 5 OR 5 OR 6 OR 7 OR 8	1,343,784
	Stent structure	
10	“structure ti,ab” OR “design ti,ab” OR “strut ti,ab” OR “strut spacing ti,ab” OR “strut thickness ti,ab” OR “open cell design ti,ab” OR "closed cell design ti,ab”	4,341,522
11	9 AND 10	17,375
12	“coronary ti,ab”	884,389
13	11 AND 12	3,191
	Search limitations	
14	Limited to English language	3124
15	Limited to human	2392
16	Limited to full text	356
17	Duplicates removed	269

The right column indicates the number of papers that each search term generated. Number 11 indicates a search command where a search term from 1 to 8 must be searched for with one of the search terms in number 10. The removal of duplicates was an automatic process performed by the Ovid tool

**Table 2:** A summary of clinical outcomes

Study	Stent type	No. of Ps	In-stent thrombosis	In-stent restenosis	TVR (%)	Anti-coagulation protocol
[24]	BRS	60	1	0	1.7	Not given
[25]	BRS	–	–	–	–	Not given
[26]	DES	–	15	0	–	Not given
[27]	BRS	90	2	0	–	Clopidogrel, prasugrel, ticagrelor
[28]	PES	–	–	–	–	Clopidogrel (75 mg) for 12 months
[29]	BMS	32	–	–	–	Clopidogrel (75 mg) for 12 months
	PES	93	–	–	–	
[30]	PES	30	0	0	–	Clopidogrel (75 mg) for 12 months
[31]	BES	369	6	–	–	DAPT for 12 months
	EES	1178	13	–	–	
[23]	SES	1261	5	–	4.1	DAPT for 12 months
	BES	1264	15	–	5.2	
[32]	BES	765	2	–	5.0	Prasugrel for 12 months
	EES	765	5	–	4.7	
	BMS	761	5	–	10.4	
[33]	DES	958	4	9	–	DAPT for 12 months
	ZES	961	6	7	–	
[34]	EES	846	3	–	3.8	DAPT for 12 months
	EES	838	5	–	3.6	
[35]	DES	82	–	82	–	Not given
[36]	SES	32	–	–	–	Not given
[37]	SES	250	–	17	7.2	Clopidogrel (75 mg) for 12 months
	BMS	250	–	52	18.8	
[38]	BRS	–	–	–	–	Not given
[39]	BMS	222	1	–	9.5	Clopidogrel (75 mg)
	PES	161	1	–	1.9	
[40]	SES	41	0	4	26.7	Clopidogrel (75 mg) for 6 months
	PES	41	0	15	8.7	
[21]	BRS	184	0	–	5.2	DAPT for 6 months (minimum)
[22]	BRS	1296	9	–	4	DAPT for 1 year (minimum)

The table displays quantitative results from the included literature including the number of cases of in-stent thrombosis, in-stent restenosis, target-vessel revascularisation (TVR), as well as the anti-coagulation protocol used

**Table 3:** The summary of data collected through the literature search: study design, stent type, drug eluted and the concentration (µg/mm^2^), scaffold material used, polymer used, stent strut thickness (µm), number of participants, gender (% male), and age (years) using means and standard deviations (SD), as well as interquartile ranges (IQR)

Study	Study design	Stent type	Drug eluted (µg/mm^2^)	Scaffold used	Polymer used	Strut thickness (µm)	Participant number	Male (%)	Age (year) (mean ± SD)
[24]	Prospective, single-arm, open-label trial	BRS	Sirolimus (3.9)	Cobalt–chromium alloy	PDDLA	80	60	73.3	67.2 ± 9.9
[25]	Cohort	BRS	Everolimus (not given)	–	PLL and PDLL	(Not given)	14	79.0	59 ± 10
[26]	Retrospective, autopsy study	DES	Sirolimus and paclitaxel (not given)	Stainless steel	–	(Not given)	80	67.5	60.0 ± 12.0
[27]	Cohort	BRS	Everolimus (not given)	–	Semi-crystalline PLA	150	90	79.0	59.0 ± 10.0
[28]	RCT	PES	TAXUS Liberte-Paclitaxel (3.9)	Stainless steel	Durable	(Not given)	19	78.9	63.1 ± 8.2
			JACTAX (HD)-Paclitaxel (9.2)	Stainless steel	Polylactide	98	21	75.0	64.1 ± 8.7
			JACTAX (LD)-Paclitaxel (5.0)	Stainless steel	Polylactide	98	20	90.5	66.0 ± 6.6
[29]	RCT	BMS	–	Stainless steel	–	(Not given)	32	75.9	68.6 (IQR = 58.4–71.9)
		PES	TAXUS express-paclitaxel (not given)	Stainless steel	SIBS	140	93	74.2	60.4 (IQR = 53.1–69.1)
[30]	Prospective, single-arm trial	PES	Paclitaxel (not given)	(Not given)	(Not given)	(Not given)	30	76.0	67.8 ± 9.6
[31]	Cohort	BES	Biolimus (not given)	Stainless steel	PLA	112	369	78.6	62.3 ± 0.7
		EES/ZES	Everolimus/Zotarolimus	Cobalt–chromium alloy	Biocompatible	81	1178	79.4	62.8 ± 0.3
[23]	RCT	SES	Sirolimus (1.4)	Silicone-carbide	PLLA	60–80	1261	74.9	66.1 ± 10.7
		BES	Biolimus (15.6)	Stainless steel	PLLA	120	1264	75.2	64.8 ± 10.8
[32]	RCT	BES	Biolimus (not given)	Stainless steel	PLA	112	765	80.0	62.0 ± 11.0
		EES	Everolimus (not given)	Cobalt–chromium alloy	Durable	81	765	78.0	62.0 ± 11.0
		BMS	–	Silicone-carbide	–	60	761	75.0	63.0 ± 11.0
[33]	RCT	DES	Ridaforolimus (1.1)	Cobalt alloy	PBMA	87	958	78.3	63.7 ± 10.2
		ZES	Zotarolimus (1.6)	Cobalt alloy	Biocompatible	91	961	81.9	63.1 ± 10.3
[34]	RCT	EES	Everolimus (1.0)	Platinum–chromium alloy	PLGA	74–81	846	70.6	63.5 ± 10.4
		EES	Everolimus (1.0)	Platinum–chromium alloy	PBMA	81–84	838	72.7	63.9 ± 10.5
[35]	Retrospective, autopsy study	DES	Sirolimus, paclitaxel or everolimus (not given)	(Not given)	(Not given)	(Not given)	82	65.9	58.3 ± 11.0
[36]	Cohort	SES	Sirolimus (not given)	(Not given)	(Not given)	140	32	93.8	65.2 ± 8.2
[37]	RCT	SES	Sirolimus (1.8)	Stainless steel	PBMA	140	250	78.0	67.4 (IQR = 59.0–75.4)
		BMS	–	(Not given)	–	76	250	78.0	66.7 (IQR = 59.9–74.7)
[38]	Cohort	BRS	Everolimus (1.0)	PLLA	PLLA	157	56	71.0	60.0 ± 8.0
[39]	RCT	BMS	–	Cobalt–chromium alloy	–	65	161	72.7	69.1 ± 12.7
		BMS	–	Stainless steel	–	80	222	75.7	69.8 ± 11.5
[40]	RCT	SES	Sirolimus (2.0)	(Not given)	PE/PLA	(Not given)	46	85.0	66.8 ± 9.5
		PES	Paclitaxel (0.25)	(Not given)	PE/PLA	(Not given)	45	89.0	67.3 ± 8.6
[21]	Prospective, single-arm trial	BRS	Sirolimus (1.4)	Absorbable magnesium mixed with rare earth metals	PLLA	150	184	63.0	65.5 ± 10.8
[22]	RCT	BRS	Everolimus (not given)	PLLA	PLLA	156	1296	72.0	63·1 ± 10·1

*RCT* randomised control trial, *BRS* bioresorbable stent, *DES* drug-eluting stent, *PES* paclitaxel-eluting stent, *BMS* bare-metal stent, *BES* biolimus-eluting stent, *EES* everolimus-eluting stent, *ZES* zotarolimus-eluting stent, *SES* sirolimus-eluting stent, *HD * high dosage, *LD* low dosage, *PLLA* poly-l-lactic acid, *PDLLA* poly-d,l-lactic acid, *PLA* poly-lactic acid, *SIBS* styrene isoprene butadiene. *PBMA* poly-*N*-butyl methacrylate, *PLGA* poly(d,l-lactide-*co*-glycolide), *PE* polyester

### Coronary stent structure

As a foreword, it is important to consider developments in coronary imaging technology, including magnetic resonance angiography (MRA) and rotational X-ray angiography [41, 42]. This allows for more accurate coronary imaging, detection and identification of pathologies. This could be decisive when choosing between PCI or CABG for the treatment of CAD.

Although there have been great advances in stent development, the basic structure remains the same. Results of recent studies highlight the importance of structure, design and material optimisation of stents [43, 44]. A metal, metal alloy or polymer scaffold is made up of struts and assembled into a particular pattern (cell design). These struts can vary in thickness and number, which are thought to be factors affecting re-endothelialisation [18]. Each structural property presents a large variation with different physiological issues [45]. The current solution is a compromise between stent function and high associated risks of ISR and IST. Vast improvements are still needed.

The current increased rates of ISR and IST in current stent designs need to be further investigated to understand the factors affecting such complications. This review, therefore, aims to investigate past and current stent design as well as the associated physiological consequences. This will be achieved by systematically searching databases to identify the relevant literature. This should help form a summary of favourable properties that need to be considered in order to design a stent that incorporates solutions to current issues whilst still being effective as a vascular scaffold in the treatment of CAD.

## Results

### Literature search and selection process

**Fig. 1:**
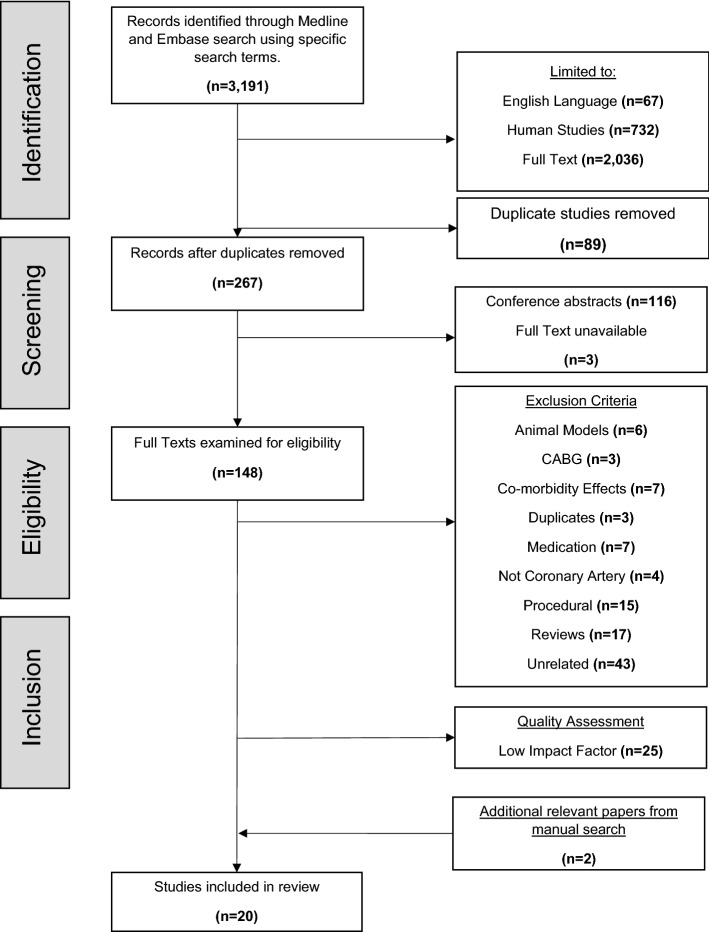
The systematic review diagram, based on PRISMA guidelines, used in this study representing the number of papers (*n*) excluded during the search process. Duplicates (*n* = 89) were removed by the Ovid software. Duplicates (*n* = 3) were removed by the single reviewer upon examination of full texts. *CABG* coronary artery bypass graft

Figure 1 summarises the literature search and selection process undertaken. From the initial search, 3191 papers were identified. Once these had been limited to those written in the English language, 67 papers were excluded, with a further 732 papers excluded as they were not human studies. Thus, 2392 papers remained. Of these, only 356 had full texts available through the Ovid program. Once duplicates had been removed by the Ovid program (*n* = 87), 269 papers remained. Conference abstracts and inaccessible full texts were removed upon attempting to access full texts (*n* = 119). In total, 148 full texts were examined for eligibility and 130 were excluded based on the exclusion criteria. The quality of full texts examined was assessed, excluding 25 papers due to low impact factors, leaving 18 papers. An additional 2 papers were included from a manual search, bringing the paper total to 20 for this review.

### Literature search results

**Fig. 2:**
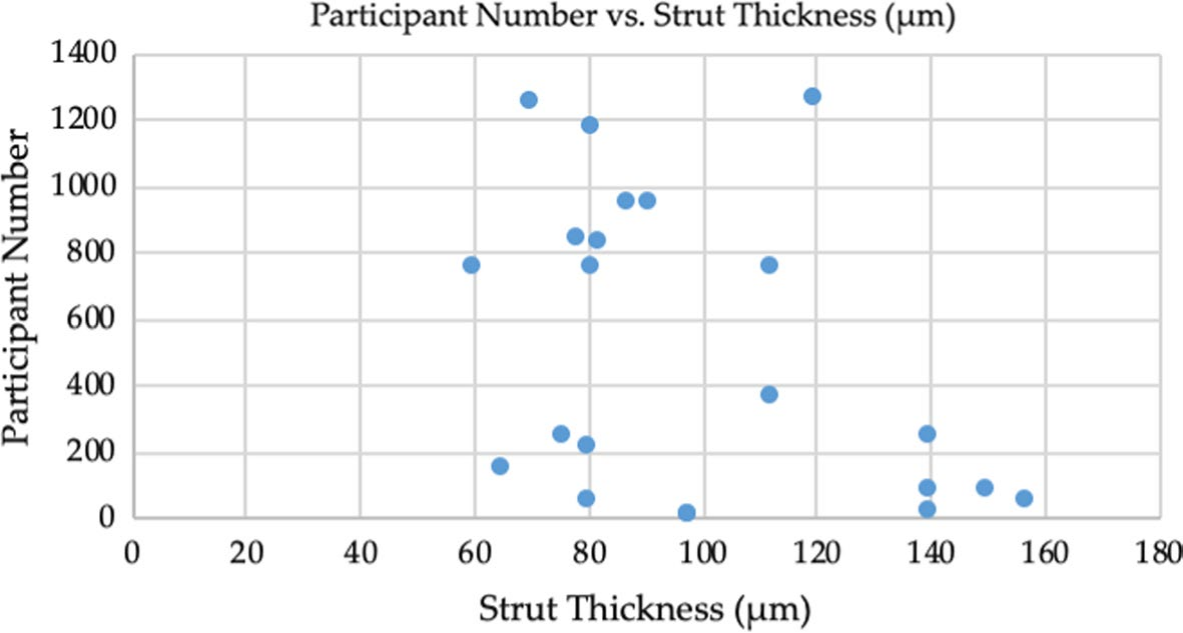
A scatter plot showing the data for participant number and strut thickness (µm)

Table 1 summarises the data collected from the included studies. Ten of the 20 studies were randomised control trials (RCT) [22, 23, 28, 29, 32–34, 37, 39, 40], five were cohort studies [25, 27, 31, 36, 38], two were retrospective autopsy studies [26, 35], and three were prospective, single-arm trials [22, 24, 30]. Four studies looked at BMS, with one comparing two BMS with varying strut thickness [39], and three comparing the effects of BMS and DES on ISR rates [29, 32, 37]. Six studies investigated BRS [21, 22, 24, 25, 27, 38], and 19 out of the 20 included studies investigated at least one drug-eluting stent. There were missing data for the scaffold and polymer types used for some studies as well as strut thickness. Eight studies included stents using stainless-steel scaffolds [23, 26, 28, 29, 31, 32, 37, 39], with five using cobalt alloys [24, 31–33, 39], one using platinum–chromium alloys [34], two using silicone-carbide scaffolds [23, 32] and one using absorbable magnesium mixed with rare earth metals [21]. Strut thickness varied between 60 µm [31, 32] and 157 µm [27]. The number of participants varied between the studies, with some studies having as few as 14 participants [25] and some as many as 1296 [22]. Figure 2 shows a scatter plot of the study sample sizes and strut thicknesses. Of these participants, on average 77.4% were male, with a mean age of 64 years.

### Outcome measurements

#### Neointima thickness and strut coverage measurements

The included studies differed in how investigators measured neointima thickness within the stented area, although many used a combination of imaging modalities. Intravascular Ultrasound is an imaging modality that uses sound waves to generate images with a resolution of ~ 100–200 µm that can be analysed to determine neointima thickness as well as strut coverage (re-endothelialisation) [46]. Intravascular ultrasound was used in five studies [23, 26, 29, 34, 38]. Intravascular optical coherence tomography is a high-resolution (~ 10–20 µm) and contrast-imaging modality. Optical coherence tomography uses infrared light to distinguish between tissues, such as endothelium and neointima, which makes it an appropriate tool to measure neointima thickness accurately. Optical coherence tomography was used by seven studies investigating neointima hyperplasia or strut coverage [23, 26, 27, 29, 31, 32, 38]. Optical frequency domain imaging is a form of optical coherence tomography that provides a higher resolution image and was used in one study [30, 46]. In the two autopsy studies, histological staining was conducted on slices of the stented artery to determine the presence of neointima as well as percentage strut coverage [24, 35].

#### In-stent restenosis

Of the 20 included studies, seven investigated ISR as an outcome, with seven using coronary angiographies as an outcome measure [25, 26, 29, 33, 34, 39, 40]. Coronary angiography is an imaging modality that uses X-rays and a radio-opaque substance that is intravenously administered to highlight the coronary arteries to determine any narrowing (restenosis) [47]. One study used a combination of coronary angiography, optical coherence tomography and intravascular ultrasound to detect ISR [26]. An autopsy study used histological staining to measure the restenosis of stented arteries [24]. Table 2 summarises the quantitative results of ISR, IST, target-vessel revascularisation (TVR) as well as the anticoagulation protocol used in each study. Target lesion revascularisation (TLR) was defined as any PCI/CABG intervention due to restenosis in a stented artery and was used as a measure of ISR. Similarly, TVR was defined as CABG or repeated PCI performed in the same target vessel. As a point of interest, angiography relies on accurate segmentation of the desired structure for the imaging technique to be useful. Novel solutions based on a multiscale algorithm that automatically segments coronary arteries [48], or the use of locally connected filters [49], create potential for data of higher accuracy to be gleaned from angiographies.

#### In-stent thrombosis

Thirteen studies investigated in-stent thrombosis as an outcome, five of which used optical coherence tomography [27, 29, 31, 32, 38], eight used coronary angiography [29, 31, 33, 34, 36, 37, 40, 45], three used IVUS [29, 34, 38], one used optical frequency domain imaging [30] and one used digital imaging [35]. Four studies used a combination to determine IST as an outcome [29, 31, 34, 38]. The Academic Research Consortium’s definition of IST was used by five studies [33, 34, 36, 37, 45]. This consisted of an angiographic confirmation of IST, with the thrombus originating in the stent or 5 mm proximal or distal to the stent; associated with ischemic symptoms [50].

### Quality assessment

Table 4 summarises the quality assessment conducted on the reported literature. Eighteen out of twenty included studies were in the first quintile of the impact factor score [51]. Bias was also assessed, with eight studies having no detectable bias in their methodology based on the highlighted domains. However, eight studies showed signs of selection bias [23, 24, 30–32, 35, 40, 45], four had signs of detection bias [27, 29, 35, 38], and four had performance bias [24, 33, 35, 40]. Three studies had more than one subject of bias based on their methodology [35, 38, 40].

**Table 4:** A summary of quality assessment

Study (author)	Journal	Impact factor quintile	Bias domain
[24]	Circulation: Cardiovascular Interventions	Q1	Selection bias
[25]	Circulation: Cardiovascular Interventions	Q1	Detection bias
[26]	Circulation	Q1	Selection bias, performance bias, detection bias
[27]	European Heart Journal	Q1	Selection bias
[28]	Circulation: Cardiovascular Interventions	Q1	Detection bias
[29]	Circulation	Q1	Selection bias
[30]	Circulation: Cardiovascular Interventions	Q1	Selection bias
[31]	Catheterization and Cardiovascular Interventions	Q1	Selection bias
[23]	Circulation: Cardiovascular Interventions	Q1	–
[32]	Circulation	Q1	Performance bias
[33]	Circulation	Q1	–
[34]	Circulation: Cardiovascular Interventions	Q1	–
[35]	European Heart Journal	Q1	Selection bias, performance bias
[36]	European Heart Journal	Q1	Detection bias
[37]	European Heart Journal	Q1	–
[38]	Journal of the American College of Cardiology	Q1	–
[39]	Journal of Interventional Cardiology	Q2	Selection bias, performance bias
[40]	European Heart Journal	Q1	–
[21]	Cardiovascular Revascularization Medicine	Q2	–
[22]	Journal of the American College of Cardiology	Q1	–

The table displays the included searched literature, with the journal each paper is cited from as well the associated impact factor quintile (Q1 = First Quintile, Q2 = Second Quintile), from the Scimago Journal and Country Rank [22]. Bias domains were determined by examination of study design and methodology of each paper [24]

## Discussion

This was a systematic review of 20 randomised control trials, cohort studies, and single-arm trials. This review investigated the association between coronary stent type and structure, and the risk of clinical complications, namely in-stent restenosis and in-stent thrombosis.

### In-stent restenosis

A randomised control trial conducted in 2011 compared a BMS and a paclitaxel-eluting stent (PES) to investigate the effects on clinical outcomes such as ISR risk. Patients were blindly randomised to receive either a stainless-steel BMS (*n* = 32) or a stainless-steel DES which eluted paclitaxel through a Styrene Isoprene Butadiene polymer matrix (*n* = 93). Baseline patient characteristics were similar between the two groups (75.9% male vs 74.2% male; median (interquartile range (IQR)): 68.6 years (58.4–71.9), 60.4 years (53.1–69.1), respectively) [32], which is representative of the population [52]. Cross-sectional percentage stenosis was measured using optical coherence tomography at a 13-month follow-up and found the BMS group to have a significantly higher rate of stenosis compared to the paclitaxel-eluting stent (PES) group (mean ± SD%) 57.0 ± 19.4 and 41.7 ± 20.6 (*p* = 0.0005). This difference may be due to paclitaxel inhibiting the neointimal hyperplasia of the vascular smooth muscle cell in the arterial wall. This study demonstrates the superiority of DES in reducing the rates of restenosis.

A larger randomised control trial confirmed these findings, comparing a thin-strut BMS to a sirolimus-eluting stent (SES) [39]. They concluded that SES reduced the risk of ISR, as the rate of restenosis was significantly higher in the BMS group (*n* = 250) compared to the SES group (*n* = 250), 25.5% vs 17% (*p* < 0.001), respectively. However, the lesions in the BMS group were more complex than those in the SES, which may have affected the restenosis rates, therefore future work should consider the complexity of disease when randomising participants. Comparing these two RCTs, patient characteristics were similar between the two studies, making the results more comparable and increasing the reliability of the smaller study. One explanation behind these significant differences in ISR rates between BMS and DES is due to the effects of the eluted drugs. Paclitaxel and sirolimus inhibit the proliferation of vascular smooth muscle cells; in BMS, this inhibition is absent, meaning vascular smooth muscle proliferation increases the arterial wall thickness, applying pressure to the metal scaffold, thus causing collapse and restenosis of the artery. As well as this, a vascular injury may be induced by the expansion of the stent scaffold leading to a cascade of neointima hyperplasia, resulting in restenosis [53–55]. This vascular injury is thought to be reduced significantly by the use of thinner struts, as they are thought to inflict less pressure on the arterial wall on expansion, subsequently reducing the risk of ISR [56–58].

It was noted that the BMS used in the larger trial had a thin strut (76 µm), which has been shown to affect restenosis rates. This factor may limit the comparability between the two studies as this was not stated in the smaller trial [52]. The material of the scaffold used in the stent has also been shown to play a role in restenosis, with one study finding that stainless-steel BMS had a higher rate of ISR compared to cobalt–chromium alloy BMS [40]. The trial used rates of target lesion revascularisation (TLR) as a measure of restenosis. The rates of TLR were 1.9% in the cobalt–chromium alloy group, compared with 8.6% in the stainless-steel group (*p* = 0.006). The underlying mechanism causing this significant difference between the two scaffolds remains unclear; however, the finding of this significant difference suggests stent material could further investigated in the future development of safer and more effective stents.

This study did not have a randomised allocation design and, therefore, the choice of the stent was influenced by the operator’s judgement. There was a higher rate of hospitalisation in the stainless-steel group, and it is possible that rates of TLR may have been increased in this group due to more frequent angiographies at hospital visits. The cobalt–chromium alloy stent is also thinner (65 µm) than the stainless-steel stent (80 µm). Although they are both relatively thin, previous research has suggested that thinner struts reduce the rate of ISR, meaning that the scaffold material may not be the only factor affecting the TLR rates [45]. Further work could compare two BMS with the same strut thickness, but with different scaffold materials, removing strut thickness as a confounding variable.

### Neointima hyperplasia and strut coverage

Although it is evident that DES is superior in reducing the risk of ISR when compared to BMS, there are still some drawbacks. The neointima hyperplasia-inhibiting drugs, such as sirolimus and paclitaxel, also inhibit the process of re-endothelialisation [11, 15]. This is the process of the stent struts becoming integrated within the endothelium as endothelial cells grow around the individual struts. A retrospective autopsy study found that stents with thrombi present had significantly more uncovered struts compared to stents without thrombi [35]. This demonstrates the significant impact that uncovered struts have on the risk of IST. In DES, the percentage of uncovered struts has been shown to be significantly higher when compared to BMS [32]. An RCT found that BMS has a higher rate of restenosis compared to SES. This study shows that the percentage of uncovered struts was significantly higher in the DES group when compared to BMS (mean ± SD %) (5.7 ± 7.0 vs 1.1 ± 2.5; *p* < 0.0001) [37]. The difference in the number of uncovered struts may be due to the strut thickness [45]. These findings suggest that SES is at a higher risk of IST, due to a greater number of uncovered struts, compared to BMS. In the present study, the SES strut was very thick (140 µm). Although the BMS strut thickness could not be found in the literature, it is thought that BMS struts are typically thinner due to the lack of polymer coating of the drug matrix. The two groups had similar baseline patient characteristics, removing this as a potential confounding variable. This significant difference has been seen in biolimus-eluting stents (BES) as well as SES [59].

Conversely, several studies have found favourable strut coverage of DES in SES and PES in single-arm trials [29, 30, 32, 60]. One study investigating strut coverage in PES found that ~ 90% of the neointima response and thickening occurred within the first three months and was sufficient for favourable strut coverage, with most stent struts covered at three months [60]. They concluded that at nine months, the rate of neointima hyperplasia and strut coverage was minimally different when compared to the rate at three months.

Optical coherence tomography was used to measure the neointimal coverage of the struts. Although it has a high resolution, it is not able to accurately distinguish between fibrin and neointima, questioning the validity of the results. This could suggest that the clinical implications of uncovered struts occur after nine months. This study did not have a control, and therefore this early strut coverage may also be seen in BMS [61]. Important structural properties of the stent, such as strut thickness, which could potentially influence the rate of strut coverage, were not given in the study.

Support for this study comes from another single-arm trial, with a larger sample size (*n* = 60), that investigated an SES with a cobalt–chromium alloy, with thin struts (80 µm) [30]. They used optical frequency domain imaging modality to measure strut coverage at one, two and three months and found a favourable strut coverage at three months. This study demonstrated a favourable strut coverage, suggesting a lower thrombotic risk, showing that the SES has a satisfactory safety profile at a one year follow-up [30, 35]. These two studies may not be comparable due to the difference in drug release kinetics seen between PES and SES [30]. Paclitaxel seems to be unaffected by different release kinetics, however sirolimus seems to be effective only with mid-term drug release. This difference may be a result of the drugs’ different cellular mechanisms. Paclitaxel targets the microtubule organisation of the vascular smooth muscle cell, whereas sirolimus targets the mTOR molecule [11, 15, 25]. The use of optical frequency domain imaging presents the same issues as optical coherence tomography, as although the resolution is greater in optical frequency domain imaging, compared to optical coherence tomography, the same issue remains in distinguishing between neointima and fibrin. Similarly, there was no control or comparison to BMS, and it is therefore difficult to determine the superiority of strut coverage of DES compared to BMS [61]. The conflicting results from these studies suggest that vascular smooth muscle cell inhibition is not the only factor affecting strut coverage; strut thickness may also influence this.

### Strut thickness and apposition

One RCT compared a thin-strut SES (60 µm) with a thick-strut BES (140 µm) when investigating the rates of IST. While there were no significant differences in the two groups at a 12-month follow-up, the rate of definite stent thrombosis was significantly higher in the BES group compared to the SES group (*n* = 5 vs *n* = 15, respectively, *p* = 0.03) [45]. This outcome suggests that the thicker BES had a higher rate of uncovered struts, which has been shown to be a factor in IST [35]. Thinner struts, therefore, are thought to have an endothelialisation property that is lost with thicker stent struts [56]. This study had a large sample size (*n* = 2525) which sufficiently powered the investigation of IST and had a blinded assessor during clinical end-point measures, removing potential reporting bias. A scatter plot showing a summary of study population sizes and strut thicknesses can be found in Fig. 2. Stents with thinner struts have also been associated with lower rates of neointima hyperplasia and therefore ISR, due to the reduction in vascular injury by the struts [39]. Neointima hyperplasia also plays a role in the apposition (adherence to the arterial wall) of the stent struts when implanted, which is another potential factor in IST [62].

Neointima hyperplasia has been shown to increase the rate of incomplete stent apposition (ISA) [38]. If a stent strut is well opposed to the arterial wall in the target vessel, it is suitably in-contact with it. A cohort study of 32 patients investigated the rates of ISA in an SES with a strut thickness of 140 µm, using optical coherence tomography and intravascular ultrasound [38]. Optical coherence tomography image capture detected a higher incidence of ISA relative to intravascular ultrasound, demonstrating the superiority of optical coherence tomography as an imaging modality. Thrombus was detected significantly more frequently in malapposed struts compared to well-apposed struts (20.6% vs 2.0%, respectively, *p* < 0.001). The primary mechanism for ISA is thought to be due to the inhibition of the neointima proliferation caused by the sirolimus. Although this significantly reduced the ISR rates, uncovered malapposed struts are thought to be the primary mechanism for ISA, being a key thrombogenic marker thought to increase the risk of late stent thrombosis (LST) [62]. An RCT generated similar findings when comparing a BMS and a PES within thick struts (140 µm) in 2011 [32]. Optical coherence tomography was used to identify uncovered and malapposed struts. PES had a greater number and larger distribution of uncovered and malapposed struts compared to BMS, which may increase the risk of thrombotic events. Delayed healing with PES, caused by the inhibition of the neointima proliferation, leading to uncovered struts, increases the thrombotic risk [35, 63]. A similar study, using intravascular ultrasound, corroborated these findings, indicating that LST was associated with incomplete stent apposition [64]. However, the very thick stent struts used in the PES may be the cause of the incomplete endothelialisation rather than the eluted paclitaxel, as several studies have suggested that with increased thickness comes an increased endothelialisation process time [39, 45].

Malapposition of stent struts can also lead to uneven drug distribution, which can result in varied drug concentrations in different areas of the arterial wall [24]. An autopsy study identified uneven distribution as greater inter-strut distances, which increased the risk of restenosis in DES [24]. This was due to areas with little drug elution leading to neointima hyperplasia, causing ISR. Other areas, however, have a very strong concentration leading to increased inhibition of neointimal hyperplasia, reducing the rate of endothelialisation of stent struts, and increasing the risk of IST. Further investigations are required to determine whether the eluted drug, strut thickness or a combination of factors is increasing the risk of IST.

### Bioresorbable stents and in-stent thrombosis

Advancements in stent design have led to trials investigating the use of bioresorbable stent (BRS) struts in an attempt to reduce the increased thrombotic risk associated with metal DES. Once the drug elution process is completed, the stent struts and polymer-drug reservoir are surplus to requirements [65]. A cohort study investigated the reabsorption process of the BRS struts using optical coherence tomography and intravascular ultrasound. The study concluded that the strut coverage of a poly-l-lactic acid based everolimus-eluting stent (EES) was similar to that of metallic struts [26]. The study also detected that the stent struts had lost substantial mechanical integrity as well as radial force. However, between baseline and 12-month follow-up, there was no radial displacement of struts. This reduced mechanical integrity was unsurprising due to previously reported results of porcine models using the same stent. The molecular weight of the struts decreased by 40% at 6 months and 70% at 12 months [66]. This suggests that the concept of ISR is a time-limited process that may not affect BRS. This study did conclude that the polymer-based struts, using optical coherence tomography, did not differ from observations seen in metallic-based struts. This study did not use a control, and it is therefore difficult to conclude that the presumed increase in strut coverage observed was due to the bioresorbable properties of the stent. Support for this study came from an RCT that showed that stents with bioresorbable or biodegradable-polymers were non-inferior in clinical safety and efficacy when compared to stents with durable polymers [27, 33, 37]. The two EES compared only differed in strut polymer; the bioresorbable polymer was poly (d,l-lactide-*co*-glycolide), the durable polymer was poly-*N*-butyl methacrylate. This meant that extraneous variables were limited, and the patient groups had no significant difference in baseline characteristics. Using a biodegradable polymer reduces the risk for potential strut malapposition as well as IST. Durable polymers have been shown to delay vascular healing, leading to incomplete strut coverage as well as increased inflammation and thrombosis [67]. This study was however, only patient-blind, and therefore the operators knew which patients received which stent, increasing the risk of selection bias.

Another cohort study compared the effects of bioresorbable polymer stents and durable polymer stents. A stainless-steel BES was compared with either an EES or a zotarolimus-eluting stent (ZES), made up of cobalt–chromium alloy with a durable polymer. The BES had a poly-lactic acid polymer strut [36]. The clinical end-point for this study was the rate of IST, with 369 patients assigned to the BES group and 1178 to the EES/ZES group. The rates of IST at 5 years were low amongst the groups (1.6% vs 1.9%, respectively), showing no significant difference between the two (*p* = 0.75). There was also no difference in target-vessel revascularisation (TVR), suggesting that the BES bioresorbable stent is safe compared to the EES and ZES stents for long-term rates of IST. Although stent assignment was not random, a propensity score matching was performed with a ratio of 1:3 to allow for potential confounders, and to minimise selection bias. The BES had a strut thickness of 112 µm compared to 81 µm of the EES/ZES, which might influence the findings and may potentially mean that, if strut thicknesses were similar, the BES rates of IST would be lower. The scaffold materials used are different between the stents and this may affect the rate of re-endothelialisation of the struts, leading to differences in strut coverage and potential rates of IST [40]. Previous studies investigating BMS have concluded that thinner struts have a lower rate of IST due to a higher rate of endothelialisation [39, 40]. Further work is required to investigate the effects of strut thickness of BRS on endothelialisation rates as well as IST rates.

A phase III clinical trial, BIOSOLVE III, reviewed a magnesium based BRS, DREAMS (drug-eluting absorbable metal scaffold) 2G (2nd generation) sirolimus-eluting Magmaris™ by Biotronik. This BRS is sirolimus eluting and uses a scaffold composed of absorbable magnesium mixed with rare earth metals. Polymer used is PLLA and strut thickness is 150 µm. This trial proved the success of the BRS in 184 patients, showing no incidences of IST up to 3 years. This was detected via angiographic follow-up procedures. Other clinical outcomes were similar to those already exhibited by commonly used DES. Comparing this with the IST rate of Abbott Absorb III (1.1% after 1 year) [68] shows that perhaps BRS technology should not be dismissed due to its history of high IST rates.

BIOSOLVE IV then took place, examining a much larger cohort of patients totaling 1075. IST occurred in 5 patients, 0.5% of the trial population, measured over a period of 12 months after stent implantation. Still an improvement from Absorb. The follow-up is set 5 years from this initial examination and is yet to take place, so it is hard to assess IST in the long term for this larger sample trial. One key factor that could explain this low IST rate when compared with its problematic predecessor, Absorb, is the electropolished struts. Although the strut thickness is similar, this electropolishing gives the struts smooth and rounded edges, as opposed to the sharp edges of Absorb, reducing the chance of IST [69]. Another factor reducing thrombogenicity is magnesium as the scaffold material. Magnesium is more electronegative than other metals used in scaffolds [70]. This electronegativity influences the blood in a way that reduces the chance of thrombosis.

However, stent design is not the only factor to consider regarding the successfulness of these products, the implantation procedure also has great influence, as shown by the Absorb IV trial. This tested Abbott’s Absorb BVS, which prior was withdrawn from the market due to concerns of IST incidents in the long term. This was tested in a randomised trial with better procedural technique and patient compatibility. This showed the previously underperforming BRS to have the same IST rate after 1 year (0.5%) as the Magmaris™ scaffold and widely used DES [71]. Anti-coagulation protocols should also be considered in reported IST rates as several studies used slight variations of dual anti-platelet therapy (DAPT). It could be suggested that the difference seen in IST rates between different stent structures and designs could be due to varied anticoagulation protocols. Table 2 lists the anti-coagulation protocols used within the studies.

## Conclusions

This review of stent usage in coronary angioplasty has found that drug-eluting stents (DES) are superior to bare-metal stents (BMS) with less risk of in-stent re-stenosis (ISR). Research reviewed here points to an uncertainty on which stent type is more superior in reducing in-stent thrombosis (IST), bioresorbable stents (BRS) or DES. Furthermore, strut thickness is believed to play a role in IST, with thinner struts reducing the risk. Re-endothelialisation rates are also thought to be affected by strut apposition, with malapposition leading to an increased risk of IST. The stent scaffold materials can also play a crucial role in inducing IST. Thus, cobalt alloy stents are superior to stainless-steel stents. Further clinical outcome analysis reveals that DES with cobalt–chromium alloy are safer and more efficient than their associated counterparts. However, DES still have persisting issues with uncovered struts leading to IST. Therefore, future work should focus on resolving this issue through BRS. While BRS have been shown to be as effective as DES, it is still unclear whether they are superior in effectiveness or safety, despite promising early research. Lastly, as many of these studies report varying clinical end-points, other clinical outcomes, such as target-vessel revascularization (TVR) rates remain unanswered.

Based on the findings of this review, there is a continuing need for further developments in coronary angioplasty by designing stents with structural properties that have thus far been shown to be the most desirable, such as thinner struts and bioresorbable polymers. Further work is needed to clarify the benefits of BRS in reducing the risk of IST compared to DES, as well as to investigating the effects of different scaffold materials on IST and ISR.

## Methods

### Search strategy

A literature search was conducted using MEDLINE (1946 to February 2021) and EMBASE (1974 to February 2021) between October 2020 and February 2021 using the Ovid program. The Cochrane Review guidelines were used as a template for our search strategy. The search terms used focused on two main aspects: stent type (BMS, DES, BRS) and stent structure, such as strut thickness and cell design. (Table 1 contains a full list of the search terms used.) All literature contained at least one search term for each of the two main aspects in either the title or abstract (search restricted by the use of the Boolean operator: “ti,ab”).

### Exclusion criteria

After the initial search, a number of criteria illustrated below were applied to exclude unrelated publications. They include:

- Duplicates
- Literature unrelated to search terms
- Conference abstracts
- Systematic reviews
- Inaccessible full texts
- Publication not in English
- Non-human subject studies
- Unrelated to stent design
- Stenting of non-coronary arteries.

After exclusions had been applied, the literature bibliographies were examined for potential additional articles of relevance [10].

### Assessment of bias

The quality of the included literature was assessed throughout the process of data collection using Cochrane Review quality assessment guidelines, which advise assessing for selection bias, performance bias, detection bias, attrition bias, and reporting bias within the Methodology. Selection bias was determined by how the study allocated participants to groups, such as sequence generation and allocation concealment. Performance bias was based on how the two groups differed in the treatment or care received during the study, such as the blinding of participants and investigators. Detection bias was determined by the outcome measures and whether the assessors were blinded to the groups. Reporting bias was based on which results were reported and whether some results were excluded from the report due to the nature of the findings. This assessment was not included within the exclusion criteria, however potential biases were considered during the analysis of findings. The quality of the literature was also assessed based on the impact factor of the journal from which the paper was cited. The impact factor was deemed low if the journal presenting the literature had an impact factor lower than the second quintile of the Scimago Journal and Country Rank, which is based on the number of citations per paper per year (latest data from 2017) [28].

## Data Availability

The datasets used and/or analysed during the current study are available from the corresponding author on reasonable request.

## Abbreviations

BES: Biolimus-eluting stent
BMS: Bare-metal stent
BRS: Bioresorbable stent
CABG: Coronary artery bypass graft
CAD: Coronary artery disease
DAPT: Dual anti-platelet therapy
DES: Drug-eluting stent
EES: Everolimus-eluting stent
HD: High dosage
IQR: Inter-quartile range
ISA: Incomplete stent apposition
ISR: In-stent restenosis
IST: In-stent thrombosis
LD: Low dosage
LST: Late stent thrombosis
PCI: Percutaneous coronary intervention
PES: Paclitaxel-eluting stent
RCT: Randomised control trial
SD: Standard deviation
SES: Sirolimus-eluting stent
TLR: Target lesion revascularisation
TVR: Target vessel revascularisation
ZES: Zotarolimus-eluting stent
